# IL-8 Secreted from M2 Macrophages Promoted Prostate Tumorigenesis via STAT3/MALAT1 Pathway

**DOI:** 10.3390/ijms20010098

**Published:** 2018-12-27

**Authors:** Tingjin Zheng, Guoxing Ma, Mingqing Tang, Zhongwan Li, Ruian Xu

**Affiliations:** 1 Engineering Research Center of Molecular Medicine, Ministry of Education, Fujian Provincial Key Laboratory of Molecular Medicine, School of Medicine, Huaqiao University, Xiamen 361021, China; 1601116004@hqu.edu.cn (T.Z.); gxma@tstc.edu.cn (G.M.); Tmq@hqu.edu.cn (M.T.); 1511316007@hqu.edu.cn (Z.L.); 2 Organization Department, Tangshan Normal University, Tangshan 063500, China

**Keywords:** prostate cancer, M2 macrophage, MALAT1, IL-8, STAT3

## Abstract

Prostate cancer (PCa) is a major health problem in males. Metastasis-associated with lung adenocarcinoma transcript-1 (MALAT1), which is overexpressed in PCa tissue, is associated with physiological and pathological conditions of PCa. M2 macrophages are major immune cells abundant in the tumor microenvironment. However, it remains unknown whether M2 macrophages are involved in the effects or not, and molecular mechanisms of MALAT1 on PCa progression have not yet been comprehensively explored. Here we reported that, M2 macrophages (PMA/IL-4 treated THP1) induced MALAT1 expression in PCa cell lines. Knockdown MALAT1 expression level in PCa cell lines inhibited cellular proliferation, invasion, and tumor formation. Further mechanistic dissection revealed that M2 macrophages secreted IL-8 was sufficient to drive up MALAT1 expression level via activating STAT3 signaling pathway. Additional chromatin immunoprecipitation (ChIP) and luciferase reporter assays displayed that STAT3 could bind to the MALAT1 promoter region and transcriptionally stimulate the MALAT1 expression. In summary, our present study identified the IL-8/STAT3/MALAT1 axis as key regulators during prostate tumorigenesis and therefore demonstrated a new mechanism for the MALAT1 transcriptional regulation.

## 1. Introduction

Prostate cancer (PCa) is a major health problem in men. During the past decade, therapeutic progress in PCa includes the approval of radiation therapy and either androgen-deprivation therapy or anti-androgen therapy prolongs survival among some patients with an intact prostate [1]. Despite these therapeutic advances, PCa remains the second leading cause of morbidity and is the third leading cause of male cancer death in the western world [1,2,3]. Recent studies suggested that PCa could eventually occur due to multiple mechanisms, among which tumor microenvironment (TME) had been gradually recognized as a key contributor for cancer progression, epithelial-mesenchymal transition (EMT) of the cancer cells, angiogenesis, cancer metastasis, and development of drug resistance [4,5,6,7,8].

Many groups of stromal cells, including angiogenic vascular cells, infiltrating immune cells, and cancer-associated fibroblastic cells, have been demonstrated infiltrating in TME [9,10]. Among them, macrophages are considered as one of the major abundant immune cells in the TME [7,11]. Macrophages are of two different polarization types, including the ‘classically’ activated (M1) and the ‘alternatively’ activated (M2) types. Comito’s group reported that increasing infiltration of M2 macrophages was associated with worse pathological characteristics and poor prognosis of PCa [12], while Shi’s group showed that M2 macrophages increased proliferative, migratory and invasive abilities of PCa cells [13], hinting that M2 macrophages may play an important role in PCa progression. M2 macrophages could release some factors and enzymes, such as interleukin-6 (IL-6), interleukin-8 (IL-8), transforming growth factor-β (TGF-β), matrix metallopeptidase-2 (MMP-2), and matrix metallopeptidase-9 (MMP-9) to promote angiogenesis, tumourigenesis, and metastasis [14,15]. Notably, IL-8, a CXC inflammatory chemokine, has been demonstrated to induce angiogenesis and promote the progression of many cancers. Clinical studies have also reported that the expression level of IL-8 in the serum of PCa patients increased over that of normal subjects or patients with benign prostatic hypertrophy [16]. Thorpe and colleagues also certified that stromal cells produced the IL-8 into the PCa environment and therefore increasing the metastatic properties of PCa cells [17].

Metastasis-associated with lung adenocarcinoma transcript-1 (MALAT1), also known as nuclear-enriched transcript 2 (NEAT2), is an evolutionarily highly conserved lncRNA. MALAT1 was frequently overexpressed and performed as an oncogene in several human tumor entities, including lung, breast, pancreas, colon, and liver [15,18,19,20,21,22,23]. Recently, Ren and colleagues showed that MALAT1 was overexpressed in PCa tissues and associated with the prognosis of PCa [24]. Moreover, MALAT1 overexpression was found to promote the PCa progression and appeared to be a new therapeutic target for the treatment of PCa [24,25,26,27]. Nevertheless, the key point that tumor microenvironment is involved in the effects and molecular mechanisms of overexpressed MALAT1 on PCa progression or not has not yet been comprehensively explored. Here, we revealed that M2 macrophages triggered the MALAT1 overexpression in IL-8/STAT3 dependent manner and thus promoted prostate tumorigenesis. First, we observed that M2 macrophages increased overexpression level of MALAT1, and promoted tumor progression in PCa. Further investigation showed that IL-8 secreted form M2 macrophages induced the expression of MALAT1. Moreover, we found that STAT3 served as an indispensable transcription factor in IL-8 mediated MALAT1 expression by directly binding to the MALAT1’s promoter. Together, this study demonstrated that MALAT1 overexpression modified by M2 macrophages promoted prostate tumorigenesis via STAT3/MALAT1 pathway.

## 2. Results

### 2.1. M2 Macrophages Driven From THP-1 Cells Promoted the PCa Tumorigenesis

**Figure 1:**
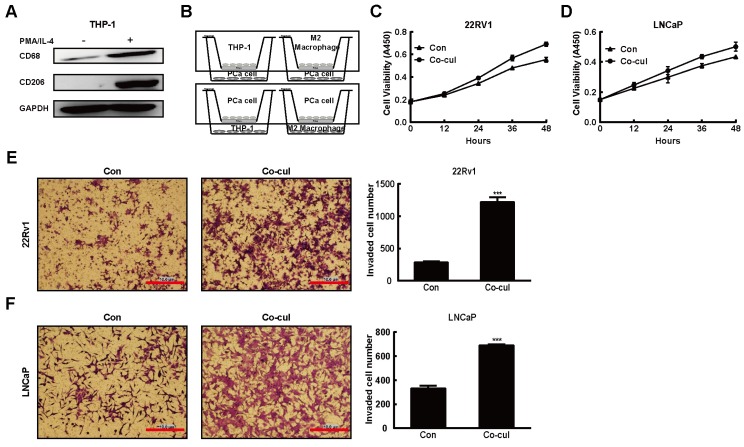
M2 macrophages promoted the prostate cancer (PCa) tumorigenesis. (**A**) PMA/IL-4 treatment induced CD68 and CD206 overexpression in THP-1 cell. Western blot was performed to assess CD68 and CD206 expression levels in THP-1 macrophages after treated with PMA (200 nM, 72 h) plus IL-4 (20 ng/mL, 36 h), with GAPDH as a loading control. (**B**) A cartoon showing the co-cultured experiment designed. (**C**,**D**) M2 macrophages promoted PCa cells proliferation. 22Rv1 (**C**) and LNCaP (**D**) were co-cultured with M2 macrophages, the proliferation of PCa cells was determined by CCK8 assay. (**E**,**F**) M2 macrophages promoted PCa cell invasion. Invasion of the 22Rv1 (**E**, upper layer) and LNCaP (**F**, upper layer) to M2 macrophages (lower layer) after incubation for 24 h was investigated. Membranes were stained with crystal violet solution and the average numbers of migrated PCa (22Rv1 and LNCaP) cells in randomly selected 3 areas counted under the microscope were shown. All data presented are the mean ± SD (*** *p* < 0.001) of triplicate determination from three independent experiments. Scale bar = 10 μm.

The THP-1 monocytic cell line was used for a model to generate differentiated macrophages for co-culture experiments [28,29]. During the differentiation induction process, THP-1 cells were treated with PMA and IL-4 [30]. The expression of macrophages surface marker CD68 and M2 macrophages surface marker CD206 increased dramatically in THP-1 cells, implying that THP-1 cell treated with PMA/IL-4 appeared to successfully differentiate into M2 macrophages (Figure 1A). To attest the potential role of M2 macrophages in PCa tumor progression, we co-cultured two PCa cell lines 22Rv1 and LNCaP with THP-1 or M2 macrophages for 48 h (Figure 1B, upper) [31]. As shown in Figure 1C, D, there was an increase in the proliferation of 22Rv1 and LNCaP cell lines after co-cultured with M2 macrophages. Previous study also reported that M2 macrophages contributed to the tumor metastasis in many solid cancers, such as breast cancer [32,33]. To assess the function of M2 macrophages in PCa metastasis, matrigel invasion assays were carried out (Figure 1B, lower). As shown in Figure 1E, once 22Rv1 cells were co-cultured with M2 macrophages, their invasive abilities increased. As expected, M2 macrophages also displayed the similar effect on LNCaP cells invasion (Figure 1F).

### 2.2. MALAT1 was Potential Mediator for M2 Macrophage-Mediated Prostate Tumorigenesis

**Figure 2:**
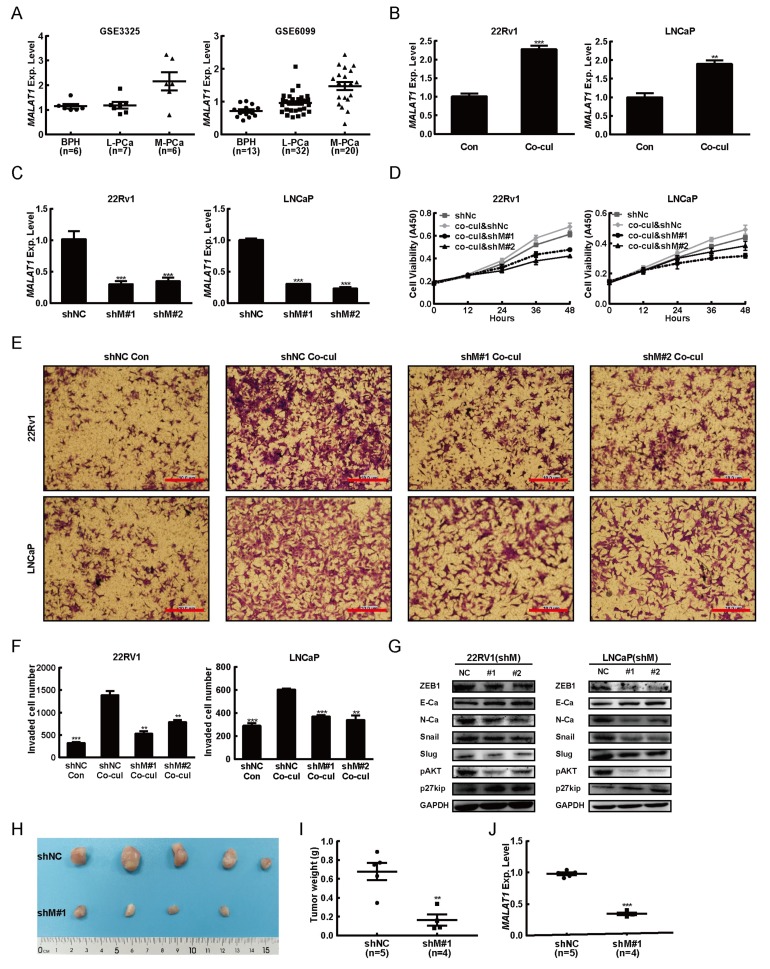
M2 macrophages up-regulated metastasis-associated with lung adenocarcinoma transcript-1 (MALAT1) promoted the PCa tumorigenesis. (**A**) Relative MALAT1 expression in benign prostatic hyperplasia (BPH), localized prostate cancer (PCa), and metastatic prostate cancer (M-PCa) tissue microarray datasets. Data sets GSE3325 (left) and GSE6099 (right) were obtained from Gene Expression Omnibus website (www.ncbi.nlm.nih.gov/geo/). (**B**) M2 macrophages induced high expression of MALAT1 in PCa cells. After co-cultured for 48 h with M2 macrophages (THP-1 as control), 22Rv1 (left), and LNCaP (right) were harvested and the total RNA of PCa cell lines (22Rv1 and LNCaP) was extracted. The level of MALAT1 mRNA was analyzed by quantitative PCR. Data presented are the mean ± SD (** *p* < 0.01, *** *p* < 0.001) of triplicate determination from three independent experiments. (**C**) Relative mRNA levels of MALAT1 in 22Rv1 (left) and LNCaP (right) cells transfected with recombinant lenti-virus expressing pLKO.1-negative control (shNC) or pLKO.1-shMALAT1s (shM#1 and shM#2) respectively. After puromycin selection, total RNA was extracted. The level of MALAT1 mRNA was analyzed by real-time PCR. Data presented are the mean ± SD (*** *p* < 0.001) of triplicate determination from three independent experiments. (**D**) Down-regulated MALAT1 expression suppressed M2 macrophages induced proliferation of PCa cells in vitro. Effect of shRNAs (shM#1 and shM#2) on 22Rv1 (left) and LNCaP (right) cells determined by CCK8 assay. Data presented are the mean ± SD of triplicate determination from three independent experiments. (**E**) Knocked down MALAT1 expression suppressed M2 macrophages induced invasion of PCa cell lines in vitro. Invasion of the PCa cell lines (22Rv1 and LNCaP, upper layer) to M2 macrophages (lower layer) after incubation for 24 h, the cells were investigated. Membranes were stained with crystal violet solution and the average numbers of invaded 22Rv1 (left) and LNCaP (right) cells in randomly selected 3 areas counted under the microscope were showed in (**F**) Data presented are the mean ± SD (** *p* < 0.01, *** *p* < 0.001) of triplicate determination from three independent experiments. (**G**) Expression levels of pAKT, p27kip, ZEB1, N-Ca, E-Ca, Snail, Slug, and GAPDH were examined by Western blot in shMALAT1s (shM#1 and shM#2) stable expressed 22Rv1 (left) and LNCaP (right) cells, respectively. (**H**) Knocked down MALAT1 expression suppressed proliferation of 22Rv1 cells in vivo. MALAT1 knockdown (shM#1) and negative control (shNC) of 22Rv1 cells were injected into the right or left flank of NCG mice (*n* = 5), respectively. After injection for 36days, all mice were sacrificed. And the excised tumors from experimental mice were representative. (**I**) Expression levels of MALAT1 was examined by real-time PCR in tumor tissues from NCG mice, respectively. (**J**) Diagram of average weight of tumors. Results presented are the mean ± SD (** *p* < 0.01,) of n determinations as reported in figure. Scale bar = 10 μm.

To clarify whether MALAT1 contributed to the M2 macrophages induced PCa tumorigenesis, we interrogated the publicly available microarray datasets derived from human benign prostatic hyperplasia (BPH), localized prostate cancer (L-PCa), and metastatic prostate cancer (M-PCa) in Gene Expression Omnibus (GEO, www.ncbi.nlm.nih.gov/geo/). We focused initially on MALAT1 expression level in two datasets (GSE3325 and GSE6099) and the data showed that the MALAT1 expression level was positively correlated to the increasing PCa degree (Figure 2A). The evidences from lab experiment also exhibited that M2 macrophages increased the expression levels of MALAT1 in PCa cell lines (Figure 2B).

To determine the biological effects of MALAT1 on M2 macrophages induced PCa progression, we down-regulated the expression of MALAT1 in the 22Rv1 and LNCaP cells by infected with lenti-virus containing shMALAT1. The outcomes showed that both 22Rv1 and LNCaP cells infected with shMALAT1 lenti-virus, appeared a lower expression levels of MALAT1 (Figure 2C). The M2 macrophages mediated cells proliferation was inhibited when the MALAT1 in PCa cell lines were silenced (Figure 2D). Consistently, the invasive ability of PCa cell lines was also suppressed while MALAT1 expression level was down-regulated by MALAT1 specific shRNAs (Figure 2E,F). In addition, knocking down expression levels of MALAT1 in PCa cell lines led to a change in the expression levels of PI3K-AKT signaling effectors pAKT and p27kip as well as the EMT markers ZEB1, N-Ca, E-Ca, snail and slug, respectively (Figure 2G). 

Our lab observations showed that both 22Rv1 and LNCaP appeared to react similarly to the stimulation of M2 macrophages, indicating that both 22Rv1 and LNCaP are reliable for tumorigenesis experiment. However, literature searching study revealed that LNCaP is much difficult for tumor formation (only 6/10 of mice grew tumor) [34]. Therefore, in this study, the 22Rv1 was run for the tumorigenesis experiment only. As shown in Figure 2H–J, knockdown expression levels of MALAT1 inhibited tumor formation of the 22Rv1 cells in vivo. All data above together demonstrated that M2 macrophages induced MALAT1 overexpression and thus promoted the PCa progression.

### 2.3. M2 Macrophages Derived IL-8 to Induced MALAT1 Expression

**Figure 3:**
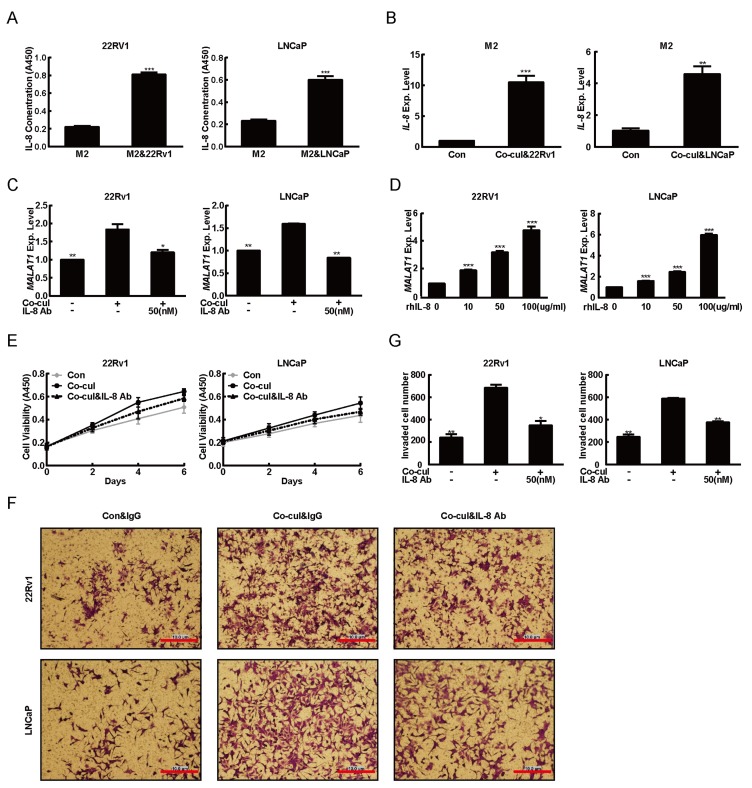
IL-8 secreted from M2 macrophages served as the stimulator for MALAT1 induction. (**A**) IL-8 concentration in supernatant of M2 macrophages culture medium was increased after cultured with PCa cells. The supernatants from the single cultures of M2 macrophages or M2 macrophages co-cultured with 22Rv1 (left) or LNCaP (right) cells were collected after incubation in serum-free medium for 24 h. The concentration of IL-8 was analyzed by ELISA assay. (**B**) IL-8 mRNA expression level in M2 macrophages was evaluated after they were co-cultured with PCa cell lines. After co-cultured with 22Rv1 (left) or LNCaP (right) cells for 48 h, M2 macrophages were harvested and total RNA was extracted (single culture of M2 macrophages was performed as the negative control). The level of MALAT1 mRNA was analyzed by quantitative PCR. (**C**) IL-8 Ab blocked M2 macrophages-induced MALAT1 expression in PCa cell lines. 22Rv1 (left) or LNCaP (right) was co-culture with M2 macrophages (normal culture was performed as the negative control), after incubation for 8 h with 50 nM IL-8 Ab (IgG was performed as the negative control) was added; After further incubation for 36 h, PCa cells were harvested for the total RNA extraction. The level of MALAT1 mRNA was measured by quantitative PCR. (**D**) rhIL-8 induced MALAT1 expression in PCa cell lines. 22Rv1 (left) or LNCaP (right) were treated with rhIL-8 (0, 10, 50 and 100 μg/mL), respectively. After incubation for 48 h, the cells were harvested for the total RNA extraction. The level of MALAT1 mRNA was measured by quantitative PCR. (**E**) Effect of IL-8 Ab on M2 macrophages-induced PCa cells proliferation was determined by CCK8 assay. (**F**) Effect of IL-8 Ab on M2 macrophages-induced PCa cells invasion was determined by trans-well assay. Membranes were stained with crystal violet solution and the average numbers of migrated PCa cells in randomly selected 3 areas counted under the microscope were showed in (**G**). All data presented are the mean ± SD (* *p* < 0.05, ** *p* < 0.01, *** *p* < 0.001) of triplicate determination from three independent experiments. Scale bar = 10μm.

Since M2 macrophages always secrete cytokines to stretch its oncogenic functions, a question arises: is any cytokine originated from M2 macrophages able to induce MALAT1 expression? Interleukin-8 (IL-8), a prototype of the cysteine-X-cysteine (CXC) chemokine, was one of the cytokines released by M2 macrophages and subsequently found to play multiple roles in cancer development [35]. Overexpressed IL-8 was also reported to promote the angiogenesis, tumorigenesis and lymph node metastasis of androgen-independent prostate cancer in athymic nude mice [36,37]. Furthermore, the IL-8 concentrations in serum appeared to correlate with the increasing PCa stages [16]. To verify whether IL-8 was involved in the PCa environment of this study or not, the IL-8 concentration in cell culture supernatants of M2 macrophages was determined after co-cultured with PCa cells. As showed in Figure 3A, there was an increased IL-8 concentration in the supernatants from M2 macrophages/PCa cells co-culture system. The IL-8 mRNA expression levels in M2 macrophages increased sharply after co-cultured with the PCa cells, implying that bidirectional communication between M2 macrophages and PCa cells pathologically promoted M2 macrophages to secrete IL-8 into tumor environment (Figure 3B). To address whether IL-8 was involved in the induction of MALTA1 expression, we added rhIL-8 and its neutralizing antibody (IL-8 Ab) into M2 macrophages/PCa cells co-culture system. The data from the lab experiments revealed that IL-8 Ab abolished the M2 macrophages induced MALAT1 expression (Figure 3C). Conversely, the expression levels of MALAT1 was induced in a rhIL-8 dose-dependent manner (Figure 3D). Subsequently, we measured the proliferative and invasive abilities of PCa cells after they were treated with IL-8 Ab, and found that the IL-8 Ab blocked M2 macrophages induced proliferation and invasion of PCa cells (Figure 3E–G). These data strongly insinuated that IL-8 secreted from M2 macrophages might play an important role in the transcriptional regulation of MALAT1 gene.

### 2.4. IL-8 Modified the Expression of MALAT1 Through STAT3 Phosphorylation

**Figure 4:**
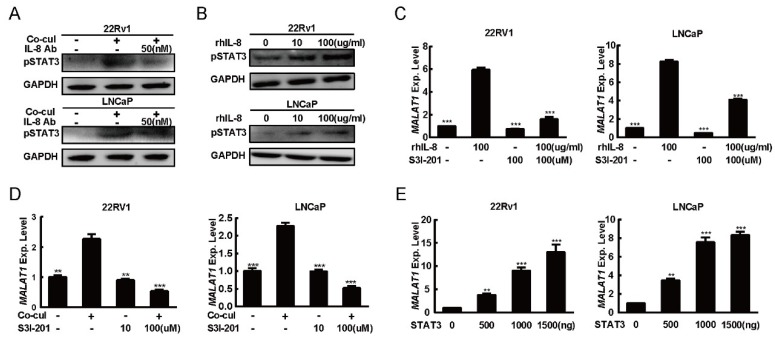
STAT3 was indispensable for IL-8 mediated MALAT1 induction. (**A**) M2 macrophages elevated the phosphorylation level of STAT3 in PCa cell lines. 22Rv1 (left) or LNCaP (right) cell line was co-cultured with M2 macrophages (normal culture was performed as the negative control). After incubation for 8 h, cells were treated with 50 nM IL-8 Ab (IgG was performed as negative control); after further incubation for 36 h, the cells were lysed and the pSTAT3 expression level was detected by western blot assay. GAPDH was applied as loading control. (**B**) rhIL-8 induced phosphorylate of STAT3 in PCa cell lines. 22Rv1 (left) or LNCaP (right) were treated with rhIL-8 (0, 10 and 100 μg/mL) respectively. After incubation for 48 h, the cells were lysed and the expression level of pSTAT3 was measured by western blot. GAPDH was applied as loading control. (**C**) STAT3 inhibitor blocked IL-8 induced MALAT1 expression in PCa cell lines. 22Rv1 (left) or LNCaP (right) cell line was treated with rhIL-8 (0 or 100 μg/mL), after incubation for 8 h, 100 µM S3I-201 (DMSO was performed as negative control) was added; after further incubation for 36 h, PCa cells were harvested for total RNA extraction. The level of MALAT1 mRNA was analyzed by quantitative PCR. (**D**).STAT3 inhibitor blocked M2 macrophages induced MALAT1 expression in PCa cell lines. 22Rv1 (left) or LNCaP (right) cell was co-cultured with M2 macrophages (normal culture was performed as negative control), after incubation for 8 h, 100 µM S3I-201 (DMSO was performed as negative control) was added; after further incubation for 36 h, PCa cells were harvested for total RNA extraction. The level of MALAT1 mRNA was analyzed by quantitative PCR. (**E**) STAT3 induced MALAT1 expression in PCa cell lines. 22Rv1 (left) or LNCaP (right) cell was transfected with pcDNA3.1- STAT3 (0, 500, 1000, and 1500 ng); at 48 h, the PCa cells were harvested for total RNA extraction. The level of MALAT1 mRNA was analyzed by quantitative PCR. All data presented are the mean ± SD (** *p* < 0.01, *** *p* < 0.001) of triplicate determination from three independent experiments.

Signal transducer and activator of transcription 3 (STAT3) signaling has been considered as a key inducer of PCa tumorigenesis [38]. STAT3 signaling has also been displayed as a downstream signaling pathways of IL-8 in many solid cancer [39,40,41]. Therefore, we investigate the phosphorylation status of STAT3 in this study. After PCa cells were co-cultured with M2 macrophages, there was an increased phosphorylation levels of STAT3, and such effect was prevented by the presence of IL-8 Ab in these cells (Figure 4A). Further data announced that the STAT3 phosphorylation levels were increased in rhIL-8 dose dependent manner (Figure 4B). In contrast, STAT3′s inhibitor (S3I-201) attenuated the rhIL-8 induced MALAT1 expression (Figure 4C). Thus, we hypothesized that STAT3 was responsible for MALAT1 expression regulation in PCa cell lines. In agreement with data above, we discovered that M2 macrophages induced MALAT1 expression was also blocked by S3I-201 (Figure 4D). To further evaluate the effect of STAT3 upon MALAT1 expression, the STAT3 expression plasmid was transfected into PCa cells. As shown in Figure 4E, MALAT1 expression levels measured by qRT PCR was elevated by STAT3 in a dose-dependent manner. All data taken together suggested that STAT3 would be responsible for the IL-8 mediated MALAT1 expression in PCa cells.

### 2.5. STAT3 Induced Expression of MALAT1 by Directly Binding to its Promoter

**Figure 5:**
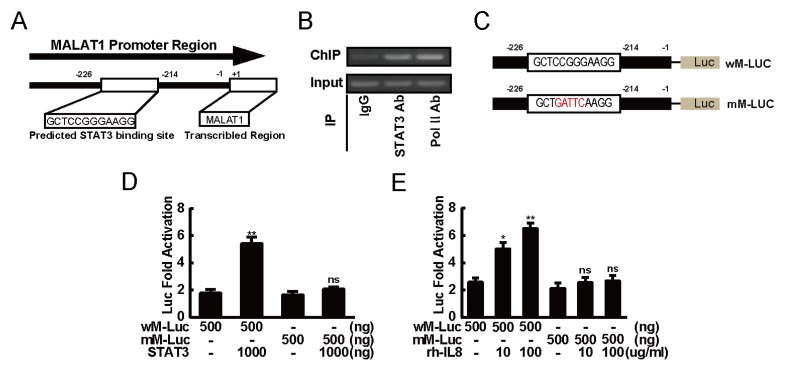
STAT3 bond to the MALAT1 promoter directly. (**A**) Structure of the MALAT1 promoter region. The region between -226 bp and -214 bp were predicted as the STAT3 binding site on MALAT1 gene promoter. +1 indicated the position of the transcription initiation site of MALAT1 gene. (**B**) ChIP assay was performed to confirm the STAT3-binding activity. Formaldehyde-cross-linked chromatin was isolated from 22Rv1 cells, sonicated, and immune-precipitated with STAT3 antibody (normal rabbit IgG was performed as a negative control, and Pol II antibody was performed as a positive control). MALAT1 DNA and 1% input was amplified using MALAT1 promoter-specific forward and reverse primers. Lane 1, immune-precipitation with normal rabbit IgG. Lane 2, immune-precipitation with the STAT3 antibody. Lane 3, immune-precipitation with the Pol II antibody. (**C**) Structure of wM-Luc and STAT3 binding site-directed mutations mM-Luc. The luciferase reporter constructs containing either STAT3 binding site or its mutated type of MALAT1 gene respectively as indicated. The positions related to the transcriptional initiation site of a MALAT1 gene (+1) as indicated. The red letter means of mutated bases in predicted STAT3 binding site. (**D**) Overexpression of STAT3-activated activity of MALAT1 promoter through the proximal STAT3-binding site. 22Rv1 cells were co-transfected with 20 ng of pRL-TK, 1 μg of pCDNA3.1-STAT3 expressing plasmid (empty pCDNA3.1 vector was performed as a negative control), along with 0.5 μg of PGL4.22 empty vector, wM-Luc or mM-Luc. After incubation for 48 h, the cells were harvested and analyzed for luciferase activity. (**E**) rhIL-8 activated activity of MALAT1 promoter through the proximal STAT3-binding site. 22Rv1 cells were co-transfected with 20 ng of pRL-TK along with 0.5 μg with PGL4.22 empty vector, wM-Luc or mM-Luc. After incubation for 8 h, rhIL-8 (0, 10 and 100 μg/mL) was added; after further incubation for 42 h, the cells were harvested and analyzed for luciferase activity. All data presented are the mean ± SD (* *p* < 0.05, ** *p* < 0.01) of triplicate determination from three independent experiments.

Since STAT3 was known to function as nuclear transcription factors by directly interacting with the promoter of its target genes and thereby contributing to start transcriptional activation in cancer. To verify the mechanisms underlying the up-regulation of MALAT1 by signaling mediated by STAT3, we made a bioinformatics analysis of MALAT1 promoter and observed that there was a potential STAT3 binding site located at -226 bp to -214 bp in the MALAT1 promoter region (Figure 5A). This indicated that STAT3 may directly bind to the MALAT1 promoter and then sparked its transcriptional activity. We performed chromatin immune-precipitation (ChIP) assay in 22RV1 cells and discovered that STAT3 was physically bond to the predicted STAT3-binding site on the MALAT1 promoter region (Figure 5B). 

To further determine the mechanism that STAT3 effects on MALAT1 transcriptional regulation, MALAT1 reporter plasmids containing wild-type or mutated STAT3-binding sites were subjected to this experiment (Figure 5C). As shown in the Figure 5D, overexpression of STAT3 could activate the wild-type MALAT1 promoter activity, but mutation of the STAT3-binding site weakly stimulated the MALAT1 promoter activity. In consistent with these observations, rhIL-8 failed to stimulate the MALAT1 promoter activity for those containing destroyed STAT3-binding site, while it could still activate the activity of wild-type MALAT1 promoter (Figure 5E). These data demonstrated that STAT3 would play an indispensable role in the transcriptional regulation of MALAT1 gene via its binding site.

## 3. Discussion

**Figure 6:**
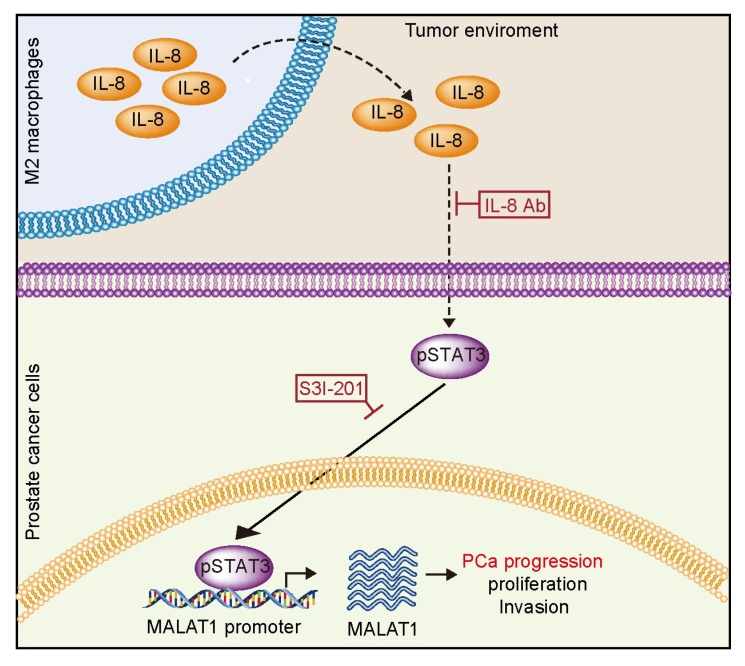
A schematic model described the mechanism of the present study that the MALAT1 expression regulation axis was modified by M2 macrophages in PCa environment. M2 macrophages secreted IL-8 into the tumor environment and thus triggered MALAT1 transcriptional activation. This activation was dependent on STAT3 and its binding site on the MALAT1 promoter reign. In addition, MALAT1 promoted the PCa progression through inducing the proliferation and invasion.

MALAT1 is overexpressed and indispensable in driving PCa cells metastasis and proliferation. Recent studies reported that MALAT1 contributed to the PCa cell invasion property through repressing the expression level of miR-145-5p and miR-1 [42,43]. Others provided evidences that MALAT1 enhanced enhancer of zeste homolog 2 (EZH2) mediated repression of polycomb dependent target genes by binding to the N-terminal of EZH2 [44]. However, the mechanism underlying overexpression of MALAT1 in PCa was poorly understood. M2 macrophages were produced during the coevolution between tumor and human immune system [45]. Moreover, numerous clinical data have shown that increasing infiltration M2 macrophages was associated with worse pathological characteristics and poor prognosis of PCa [46,47]. It is presumed that the overexpression of MALAT1 was more likely determined by the M2 macrophages. Our data of this study for first time reported that the M2 macrophages triggered the MALAT1 expression in IL-8/STAT3 dependent manner, and thus promoted the PCa progression (Figure 6).

Importantly, in this study, we clearly identified MALAT1 as a novel downstream mediator of M2 macrophages and demonstrated that silencing the MALAT1 expression could effectively block M2 macrophages mediated cell proliferation and invasion (Figure 2D–F). Consistent to the biological effects MALAT1 made on PCa, we also showed that shMALAT1 negatively affect the effector of PI3K-AKT and EMT (Figure 2G). These findings suggested a new role for MALAT1 and its downstream signaling in prostate tumorigenesis. Androgen receptor (AR) has been highlighted due to its critical role in the development of PCa. There are some studies showed that immune cells contributed to the PCa progression relayed on AR mediated signaling. Hence, androgen-deprivation therapy (ADT) is the standard therapy for men with PCa. However, ADT was also fail to achieve long-term efficacy since most PCa patients eventually relapse [9]. Recently, Wang and colleague reported that AR activity failed to regulate MALAT1 expression in 22Rv1 and LNCaP cell lines [26]. Besides, Ren’s group demonstrated the overexpression of MALAT1 in PCa cell lines, including AR negative cell lines PC-3 and DU145 [24]. These data raised the intriguing possibility that M2 macrophages could be functioning to elicit AR independent signals for MALAT1 transcriptional regulation in PCa cells during co-culture. During the tumor progression, M2 macrophages always release cytokines, growth factors or extracellular matrix proteins to achieve its oncogenic manner. IL-8, a CXC inflammatory chemokine, has been demonstrated to induce angiogenesis and to promote the progression of PCa in AR dependent and independent manner [36,48,49]. In our study, we found that IL-8 secreted from M2 macrophages induced the MALAT1 expression in PCa cells, this finding implied that AR may not be required for MALAT1 transcriptional regulation. In addition, the results of the present study displayed that the overexpression of MALAT1 in PCa cells was mediated by M2 macrophages secreted IL-8. Moreover, our data also indicated that the IL-8 expression level in M2 macrophages was mainly induced by the bidirectional communication between PCa cells and M2 macrophages (Figure 3B). It would be fascinating to determine how PCa cells modify the up-regulation of IL-8 in M2 macrophages cells precisely.

It is generally believed that the transcription of MALAT1 is initiated from multiple promoters [50,51]. Nevertheless, it is still unclear which one of these promoters is predominant and which factor regulates the selection or drive the expression so far. There were some transcription factors, including CREB, c-MYC, SP1, and SP3, reported to be involved in the MALAT1 transcriptional regulation. Wilusz and colleague have identified there was an SP1 binding site on the MALAT1 promoter and the SP1 was the domain factor to drive the MALAT1 expression in A549 [52]. In human SK-N-SH neuroblastoma cells, the cAMP response element binding transcription factor (CREB) and its binding site on the MALAT1 promoter were required for the oxytocin-mediated MALAT1 overexpression [53]. Another investigation showed that c-MYC transcriptionally activated the MALAT1 expression by physically binding to the MALAT1 promoter [54]. We predicted that there would be a STAT3 binding site on MALAT1 promoter region. Our ChIP and luciferase reporter assays also confirmed that STAT3 directly interacted with MALAT1 region, and stimulated the transcriptional activity of MALAT1 (Figure 5B,D,E). Interestingly, both STAT3 and MALAT1 over-expression occurred commonly in the tumor, and presumably such a co-occurrence might lead to tumor malignant transformation, it is likely that MALAT1 might act as a downstream effector of STAT3 in accelerating tumor progression. Coincidentally, Wang’s recent study demonstrated that STAT3 activated the MALAT1 transcriptionally activation by binding to the MALAT1 promoter region in human head and neck squamous cell carcinoma [55]. Thus, our findings provided a valuable information for understanding MALAT1 gene regulation in tumors. Nevertheless, there is still much more work need to be done in order to learn whether STAT3 mediated transcriptional activation is dominant in MALAT1 expression activation.

In summary, we have identified a novel regulatory axis that IL-8/STAT3/MALAT1 in the effort of elucidating the tumor-promoting the function of M2 macrophages. The critical effect of STAT3 on MALAT1 may shed light on how MALAT1 achieves its oncogenic manner in tumorigenesis. 

## 4. Materials and Methods

### 4.1. Cell Culture

THP-1, 22Rv1 and LNCaP cells were cultured in RPMI 1640 (Invitrogen, Gaithersburg, MD, USA) supplemented with 10% (*v*/*v*) fetal calf serum (FBS, Invitrogen, Gaithersburg, MD, USA) and antibiotics (100 units/mL penicillin, 100 µg/mL streptomycin, Beyotime Biotechnology, Nantong, China). HEK-293FT were cultured in DMEM (Invitrogen, Gaithersburg, MD, USA) supplemented with 10% fetal calf serum FBS and antibiotics. Cells were maintained in humidified 5% CO2 environment at 37 °C. 

For THP-1 differentiation, THP-1 was plated (200,000 cells/mL) in RPMI 1640 supplemented with 200 nM phorbol myristate acetate (PMA, Sigma, St. Louis, MO, USA) for 72 h to achieve macrophage differentiation. Media was then supplemented with 20 ng/mL IL-4 (Beyotime Biotechnology, China) and cultured for a further 36h prior to harvesting, to achieve polarization into M2 macrophage.

For of M2 macrophage/PCa cells (22Rv1 or LNCaP) co-culture. PCa cells were harvested and washed once with PBS. After that, the PCa cells were suspended in RPMI 1640 medium. M2 macrophage and cancer cells were cultured in the different compartment of a trans-well plate at 1:1 ratio for 48 h. 

### 4.2. Plasmids and Reagents

The sequence of STAT3 was amplified by reverse transcriptase-polymerase chain reaction (RT-PCR) and then subcloned into pDNA3.1 vector (Invitrogen, Gaithersburg, MD, USA). The putative wild-type and mutated STAT3-binding sites in the MALAT1promoter region were cloned into pGL4.22 luciferase vector (Promega, Madison, WI, USA) to contruct wM-Luc and mM-Luc; phRL-TK were purchased from Promega (Promega, USA); pLKO.1-puro, pVSV-G and pCMV-Δ8/9 were a generous gift from Bob Weinberg (#8453-#8455, Addgene, USA); Dimethyl sulfoxide (DMSO) was purchased from Sigma (Sigma, USA); S3I-201 was purchased from Selleck (Selleck, Houston, TX, USA).

### 4.3. Cell Proliferation Assay

Cell proliferation was determined using the Cell Counting Kit-8 (CCK8, Beyotime Biotechnology, China). Briefly, 2500 cells were seeded in a well of 96-well flat-bottomed plate (Corning, NY, USA). On the point of measuring the growth rate of treated cells, 100 μL of spent medium was replaced with an equal volume of fresh medium containing 10% CCK-8, cells were incubated at 37 °C for 1 h. The absorbance at 450 nm was measured using a microplate reader (Bio-Rad, Hercules, CA, USA). rhIL-8 (Novus Biologicals, Littleton, CO, USA) and its neutralizing antibody (IL-8 Ab, Proteintech, Chicago, IL, USA) was used in this study. 

### 4.4. Invasion Assay

The invasion assay was conducted using a 24-well trans-well chamber with a polycarbonate membrane with a pore size of 8 μm (Corning, NY, USA). The membrane was coated with 60 μL of 17% Matrigel (BD Sciences, San Jose, CA, USA) and serum-free RPMI 1640 medium to form a matrix barrier. After the matrigel was allowed to solidify at 37 °C for 2 h, 22Rv1 and LNCaP cells (100,000 cells/mL, treated with mitomycin C (10 μg/mL, Selleck, Houston, TX, USA) 2 h for inhibiting cell proliferation) that had or had not been co-cultured with M2 macrophage were added to the upper compartment of the chamber; the lower chamber was filled with 0.6 mL of medium supplemented with 10% FBS as a chemoattractant. After incubation at 37 °C for 24 h, Matrigel and the cells remaining in the upper chamber were removed using cotton swabs. Cells on the lower surface of the membrane were fixed in 4% paraformaldehyde and stained with Giemsa (Solarbio, Beijing, China). Cells in 3 microscopic fields were counted and photographed. All experiments were performed in triplicate. 

### 4.5. In Silico Analysis Using the Gene Expression Omnibus (GEO)

To determine the expression pattern of MALAT1 in prostate cancer, two datasets (GSE3325 and GSE6099) were downloaded from Gene Expression Omnibus (GEO) website (http://www.ncbi.nlm.nih.gov/gds/) and analyzed using the statistical language R. The MALAT1 gene expression levels in different grades of prostate cancer tissues was normalized.

### 4.6. Total RNA Extraction and Real-Time Quantitative PCR (RT-qPCR)

Total RNAs were extracted using Trizol reagent (Invitrogen, Gaithersburg, MD, USA) according to the manufacturer’s protocol, and the complementary DNA was synthesized from total RNA using Superscript III transcriptase (Invitrogen, Gaithersburg, MD, USA). RT-qPCR was performed on Step One plus system (Applied Biosystems, Foster City, CA, USA) using SYBR green (Vazyme Biotech, Shanghai, China) to determine the mRNA expression levels of interested genes. Primer sequences were as follows: MALAT1 forward primer: 5′-CGGAAGTAATTCAAGATCAAGAG-3′, reverse primer: 5′-ACTGAATC-CACTTCTGTGTAGC-3′; IL-8 forward primer: 5′-CCAACACAGAAATTATTGTAAAGC-3′, reverse primer: 5′-TGAATTCTCAGCCCTCTTCAA-3′; 18s forward primer: 5′-AACCCGTTG-AACCCCATT-3′, reverse primer: 5′-CCATCCAATCGGTAGTAGCG-3′. No amplification of nonspecific products was observed in any of the reactions, as determined from an analysis of the dissociation curves. The data were normalized to the 18s expression levels and presented as the averages from three repeated experiments. 

### 4.7. Lentiviral Transduction and Infection shRNA

pLKO.1 lenti-vector contained a hairpin sequence for homo MALAT1 specific shRNA (shM#1: 5′-TTAGCGGAAGCTGATCTCCAATGCT-3′ and shM#2: 5′-TTGAAACCGATTATGGATC-3′). For packaging of the lenti-viral vectors, 4,000,000 293FT cells were seeded in a 10 cm dish and transfected with 5 μg of pVSV-G, 10 μg of pCMV-Δ8/9 and 10 μg of transfer vector using Lipofectamine 3000 (Invitrogen, USA). At 48 h after transfection, the virus was harvested by collecting supernatants and concentrated by centrifugation at 25,000× *g* at 4 °C for 2 h.

### 4.8. In Vivo Tumorigenicity Assay

NCG mice (NOD-Prkdc^em26Cd52^Il2rg^em26Cd22^/Nju, Model Animal Research Center of Nanjing University, China) were subcutaneously injected with 1,000,000 MALAT1 knockdown (shM#1) and negative control (shNC) of 22Rv1 cells. After injection for 36 days, animals were sacrificed. Tumors weight were measured. All animals used in this study were maintained and handled according to protocols approved by the Animal Care and Use Committee of Huaqiao University (2017021, 7 November 2017–30 December 30 2018). 

### 4.9. ELISA Assay

The supernatants from the single culture of M2 macrophages or M2 macrophages co-cultured with PCa cell lines were collected after incubation in serum-free 1640 RPMI for 24 h. The medium was centrifuge do remove the cellular debris and frozen at −80 °C until subjected to ELISA to assay the levels of IL-8. The IL-8 concentrations were determined using a commercial ELISA kit (R&D Systems, Minneapolis, MN, USA) according to the manufacturer’s instructions.

### 4.10. Western Blotting

Interested cells were lysed in cold RIPA Lysis and Extraction Buffer (Invitrogen, Gaithersburg, MD, USA) containing a protease inhibitor (Selleck, Houston, TX, USA). After centrifugation, equal amounts of the protein were loaded for electrophoresis on 10% denaturing SDS-PAGE gels. The blots were probed with the primary antibodies overnight at 4 °C, followed by incubation with the appropriate horseradish peroxidase (HRP)-conjugated secondary antibodies at room temperature for 2 h. CD68 (1:1000, CST, Beverly, MA, USA), CD206 (1:1000, CST, Beverly, MA, USA), E-Ca (1:1000, CST, Beverly, MA, USA), N-Ca (1:1000, CST, Beverly, MA, USA) (1:1000, CST, Beverly, MA, USA), snail (1:1000, CST, Beverly, MA, USA), slug (1:1000, CST, Beverly, MA, USA), p27kip (1:1000, CST, Beverly, MA, USA), pAKT (1:1000, CST, Beverly, MA, USA), pSTAT3 (Tyr705, 1:1000, CST, Beverly, MA, USA) or GAPDH (1:1000, CST, Beverly, MA, USA) were used in this study.

### 4.11. Luciferase Assay

22Rv1 cells were plated on 12-well plates at 250,000 cells per well. After establishment for 24 h, cells were co-transfected with 10 ng phRL-TK and 500 ng luciferase reporter plasmid (wM-Luc or mM-Luc). To locate the function of STAT3 in MALAT1, the 22Rv1 were co-transfected with 500 ng pDNA3.1-STAT3. To locate the function of IL-8 in MALAT1, the transfected cells were treated with rhIL-8 for 42 h before being harvested for the reporter assay. At 48 h after transfection, a luciferase reporter assay was performed using dual-luciferase reporter assay system (Promega, Madison, WI, USA) according to the manufacturer’s instructions. Each experiment was performed in triplicate, and the data represent the mean plus or minus SD of 3 independent experiments, each normalized to renilla activity.

### 4.12. Chromatin Immunoprecipitation (ChIP)

Briefly, protein-DNA complexes were cross-linked by 1% formaldehyde (Sigma, St. Louis, MO, USA) for 10min, then quenched using 125 mM glycine for 5 min. After washing three times with cold PBS, cells were collected in digested buffer and digested into the purpose of chromatin DNA. After centrifugation, the supernatant was incubated with the IgG (Abcam, Cambridge, MA, USA), Anti-Pol II (CST, Beverly, MA, USA) or Anti-STAT3 (10253-2-AP, Proteintech, Chicago, IL, USA) overnight at 4 °C. Next, pre-clear A/G beads (Invitrogen, Gaithersburg, MD, USA) were added for another 1h, and chromatin DNA was purified and subjected to PCR detection. The set of primers used was as follows: forward 5′-CATGCCATTCCCCAGAACAGGC-3′ and reverse 5′-CCGCGCAGGGATACGCGA-3′. The predicted PCR product length is 192bp. 

### 4.13. Statistical Analyses

All results are presented as the mean ± standard error of mean (SEM). Statistical analysis of diferences between groups was performed using the Graphpad prism 7 software (Graphpad Software, San Diego, CA, USA), and *p* < 0.05 was considered to be significant. 

## Abbreviations

PCa	Prostate cancer
MALAT1	Metastasis-associated with lung adenocarcinoma transcript-1
EMT	epithelial-mesenchymal transition
IL-8	interleukin-8
STAT3	Signal transducer and activator of transcription 3
